# AI-based automatic segmentation of craniomaxillofacial anatomy from CBCT scans for automatic detection of pharyngeal airway evaluations in OSA patients

**DOI:** 10.1038/s41598-022-15920-1

**Published:** 2022-07-13

**Authors:** Kaan Orhan, Mamat Shamshiev, Matvey Ezhov, Alexander Plaksin, Aida Kurbanova, Gürkan Ünsal, Maxim Gusarev, Maria Golitsyna, Seçil Aksoy, Melis Mısırlı, Finn Rasmussen, Eugene Shumilov, Alex Sanders

**Affiliations:** 1 Department of Dentomaxillofacial Radiology, Faculty of Dentistry, Ankara University, Ankara, Turkey; 2 Medical Design Application, and Research Center (MEDITAM), Ankara University, Ankara, Turkey; 3 Department of Dental and Maxillofacial Radiodiagnostics, Medical University of Lublin, Lublin, Poland; 4 Diagnocat Inc., San Francisco, CA USA; 5 Department of Dentomaxillofacial Radiology, Faculty of Dentistry, Near East University, Nicosia, Cyprus; 6 Research Center of Experimental Health Science (DESAM), Near East University, Nicosia, Cyprus; 7 Internal Medicine Department Lunge Section, SVS Esbjerg, Esbjerg, Denmark; 8 Life Lung Health Center, Nicosia, Cyprus

**Keywords:** Dentistry, Diagnosis, Medical imaging, Radiography, Tomography

## Abstract

This study aims to generate and also validate an automatic detection algorithm for pharyngeal airway on CBCT data using an AI software (Diagnocat) which will procure a measurement method. The second aim is to validate the newly developed artificial intelligence system in comparison to commercially available software for 3D CBCT evaluation. A Convolutional Neural Network-based machine learning algorithm was used for the segmentation of the pharyngeal airways in OSA and non-OSA patients. Radiologists used semi-automatic software to manually determine the airway and their measurements were compared with the AI. OSA patients were classified as minimal, mild, moderate, and severe groups, and the mean airway volumes of the groups were compared. The narrowest points of the airway (mm), the field of the airway (mm^2^), and volume of the airway (cc) of both OSA and non-OSA patients were also compared. There was no statistically significant difference between the manual technique and Diagnocat measurements in all groups (*p* > 0.05). Inter-class correlation coefficients were 0.954 for manual and automatic segmentation, 0.956 for Diagnocat and automatic segmentation, 0.972 for Diagnocat and manual segmentation. Although there was no statistically significant difference in total airway volume measurements between the manual measurements, automatic measurements, and DC measurements in non-OSA and OSA patients, we evaluated the output images to understand why the mean value for the total airway was higher in DC measurement. It was seen that the DC algorithm also measures the epiglottis volume and the posterior nasal aperture volume due to the low soft-tissue contrast in CBCT images and that leads to higher values in airway volume measurement.

## Introduction

Obstructive sleep apnea (OSA) is identified by periods of partial or complete upper airway disruption during sleep. OSA patients can breathe normally when they are awake but the disruptions occur since those patients cannot preserve the pharyngeal airway space when they sleep^1,2^. OSA patients who do not receive any treatment may have hypertension, heart failure, stroke, and premature death^3^. OSA patients are unavailable to preserve the pharyngeal airway space when they sleep, however, they can breathe normally when they are awake^2,3^.

Inferior displacement of the hyoid bone, mandibular insufficiency, and increased soft palate and tongue volume are reported in the etiology of OSA in the literature^4^. Frequent reasons for collapsing of the upper airway are described as; the competence of the airway by reflexes, pharyngeal inspiratory muscle activity, and anatomic contraction of the upper airway^5,6^. Since the diagnosis of OSA requires a multidisciplinary approach, a dentist, a neurologist, a cardiologist, an otorhinolaryngologist, and a pulmonary medicine specialist should be involved in the diagnosis and the treatment process^7^.

CBCT is a 3-Dimensional radiographic diagnostic unit that can scan a region of interest with superior hard-tissue contrast and this provides a thorough analysis of the bony structures which are crucial in OSA diagnosis^8,9^. Thanks to its lower dose, lower cost, and higher image quality, CBCT is preferred over other advanced imaging methods such as Multi-Detector CT in dentistry, especially for the evaluation of the craniofacial structures^10^.

Several articles are present in the literature with specific deep learning models for automatic segmentation of the maxillofacial structures, mandibular canal, cephalometric landmarks, cervical vertebras, and maxillofacial defects such as cleft palate. The majority of these models had U-Net architecture with a high (90%-95%) Dice Similarity Coefficient^11–26^.

Anatomical structures such as the craniofacial skeleton and soft tissues which surround the muscles and pharynx have an important role in the configuration of the upper airway. Pharynx morphology is known to be one of the major factors that may cause OSA. Air-flow obstruction in children is also thought to occur due to skeletal deficiency since the contraction in the anterior–posterior aspect of the airway ensues from the positioning of the mandible and maxilla^27–29^.

Numerous software is available to analyze the CBCT data with semi-automatic or manual volumetric measurement process^30^, however, most of that software is laborious, time-consuming and a completely automatic airway detection algorithm is limited^12^. Thus, this study aimed to generate and also validate an automatic detection algorithm for pharyngeal airway on OSA patients' CBCT data using an artificial intelligence software of Diagnocat (DC).

## Materials and methods

The research protocol was performed following the principles of the Declaration of Helsinki and was approved by the non-interventional Institutional Review Board (IRB) of Near East University Health Sciences Ethics Committee (YDU/2022/87-1251). Written informed consent was obtained from all patients before their radiographic examinations and anonymization was performed in compliance with the Information Commissioner's Anonymization: managing data protection risk code of practice (https://ico.org.uk/media/1061/anonymisation-code.pdf). The study data was created only from the deidentified anonymized data.

Anonymized DICOM files of the CBCT images which were taken by 3 different CBCT units were used in this study. The CBCT units were Pax-i3D Smart PHT-30LFO0 (Vatech, South Korea), Carestream Health CS 8100 3D (Kodak, USA), and Orthophos XG 3D (Sirona, Germany). All mentioned CBCT units have isotropic voxels which differ between 0.1 and 0.2 mm^3^.

This study aimed to generate an AI algorithm for segmentation of the craniomaxillofacial anatomy and to test this algorithm for automatic detection algorithm for pharyngeal airway both for OSA and control patients. Thus, this study has two notable parts dataset preparation for the evaluation and to test the practicability of the system to enhance the diagnostic capabilities.

### CBCT anatomy localization generated with an AI model

#### Approach

To handle large volume sizes on a reasonably fine scale, we approach this task with a coarse-to-fine approach. In general, a coarse-to-fine framework performs an inference at successively finer scales. The approach uses the results from the previous coarser stages to guide and speed up inference at the finer stages. A coarse-to-fine framework allows to achieve high-quality segmentation masks while being efficient during inference.

In this model, we use a two-stage coarse-to-fine approach. Both stages are defined as semantic segmentation tasks but at different voxel scales. At the first (coarse) stage, the whole volume is analyzed at once in a single forward pass through the neural network. During this stage, the model operates in coarse resolution of scale 1 mm. The goal of this stage is to perform a coarse segmentation of anatomical structures in a computationally efficient manner.

Next, we pass the results of the first stage as an input to the second (fine) stage. The fine stage allows us to achieve accurate segmentation masks by refining the outputs of the coarse stage. In this work, the fine stage is implemented as a patch-based semantic segmentation. The main idea of a patch-based approach is to train a neural network on small parts of the original images (and not the whole images) which leads to substantially reduced required computational resources. During inference, we extract the patches from the original image with an overlap and pass them through the model one by one. The results are then aggregated to form the final segmentation masks. At this stage, the training and inference are performed on a fine voxel scale of 0.25 mm.

Our approach to training the system consists of 4 main steps which are described in detail in the following sections: preprocessing of incoming volumetric image; coarse model training; coarse hint generation; patch-based training in fine resolution with a hint from the coarse model.

#### Data

We use a simple min–max normalization within a fixed window. We clip the intensities to be inside the [− 1000, 2000] interval, then subtract a minimum intensity value and divide by a maximum one. Different methods have also been examined. According to our experiments, the training procedure is not sensitive to the choice of preprocessing and all methods lead to approximately the same results. The data is split into training, development, and test sets. We use 90% of the data for training, 5% for the development set, and 5% for the test set.

For the Coarse step, we rescale the image to have a 1.0 mm isotropic voxel resolution using linear interpolation. To provide the Coarse model with more information, we obtain soft coarse segmentation ground truth labels by the following procedure. First, we encode the original semantic segmentation mask of shape DxHxW with a one-hot encoding scheme which results in a tensor of shape tensor CxDxHxW, where C represents the number of classes and D, H, and W are the spatial dimensions of the original volume. Next, we use linear interpolation to rescale this tensor to have a 1.0 mm resolution. The resulting tensor consists of the probability distributions over classes for each spatial position and is referred to as soft targets.

For the Fine step, the target voxel spacing of the model is 0.25 × 0.25 × 0.25 mm which is also achieved with linear interpolation of the image. For this step, we obtain the ground truth labels via a simple nearest-neighbor interpolation of original semantic segmentation masks. During training, we randomly sample patches of size 144 × 144 × 144 voxels.

#### Model

We formulate both Coarse and Fine steps as a semantic segmentation task, where the background and each anatomical element are interpreted as a separate class. For both Coarse and fine steps, we use 3D U-Net^31^ which is a standard, widely known, and well-studied fully convolutional neural network architecture^14,32–37^. Our implementation follows the architecture which is described in detail in the original paper^31^.

Since the Fine model is trained using a patch-based approach, it's crucial to provide the model with global information. We achieve it by utilizing a coarse hint. A coarse hint is the Coarse model output which is interpolated to the Fine-scale and passed to the Fine model as additional input channels. To prevent possible data leakage, we train the Coarse model and prepare coarse hints via three-fold cross-validation. Therefore, the only difference between the Coarse and Fine model architectures is the number of input channels: for the Coarse step it equals 1, and for the Fine step, it equals the number of classes plus 1.

The class imbalance is known to be a challenging problem in medical semantic segmentation tasks. We approach this issue by using a sum of a standard cross-entropy loss and soft multiclass Jaccard loss. To prevent overfitting and enhance the performance of the model we also utilize a large variety of data augmentations. For the Coarse step the following augmentations are used during training: random blur, random noise, random rotations, random scaling, random crops, random elastic deformation, and random anisotropy^38^. For the Fine step, we used the same set of augmentations except for random elastic deformation and random anisotropy since these transformations are computationally expensive when applied to reasonably large images.

#### Training

To sum it up, our training procedure consists of the following steps. First, we train the Coarse model on the coarse training dataset with soft targets. This checkpoint is used during the testing. We also perform three-fold cross-validation and use the obtained checkpoints later to generate coarse hints for the Fine step. For both cross-validation and full data training, we follow the same procedure. We train for a total of 100 epochs using an Adam optimizer with a one-cycle scheduling policy with a maximum learning rate equal to 1e−3, minimum learning rate equal to 1e−6, warmup fraction of 0.05, and a batch size of 1.

Next, we prepare coarse hints for the Fine model. We utilize the checkpoints received via cross-validation and make out-of-folds predictions, then linearly interpolate the output probability maps to the Fine model voxel spacing and concatenate them with the original intensity value channel. Finally, we train the Fine model for a total of 40 epochs, using the Adam optimizer and the same learning rate scheduling policy, as in the Coarse step.

To train the Fine model we use a patch-based approach. At the beginning of the training epoch, we iterate over the images, randomly sample 20 patches per volume and store them in a queue of size 180. Once the queue has reached a specified maximum length we start to retrieve the random patches from it and pass them to the network while simultaneously preparing new patches and adding them to the queue. For evaluation, we use the checkpoint with the lowest recorded validation loss for both Coarse and Fine models.

#### Implementation

Our algorithm was based on the Python implementation of U-net. All training and experiments were done using NVIDIA GeForce RTX A100 GPU. Adam optimizer was used for the network training.

#### Inference

**Figure 1:**
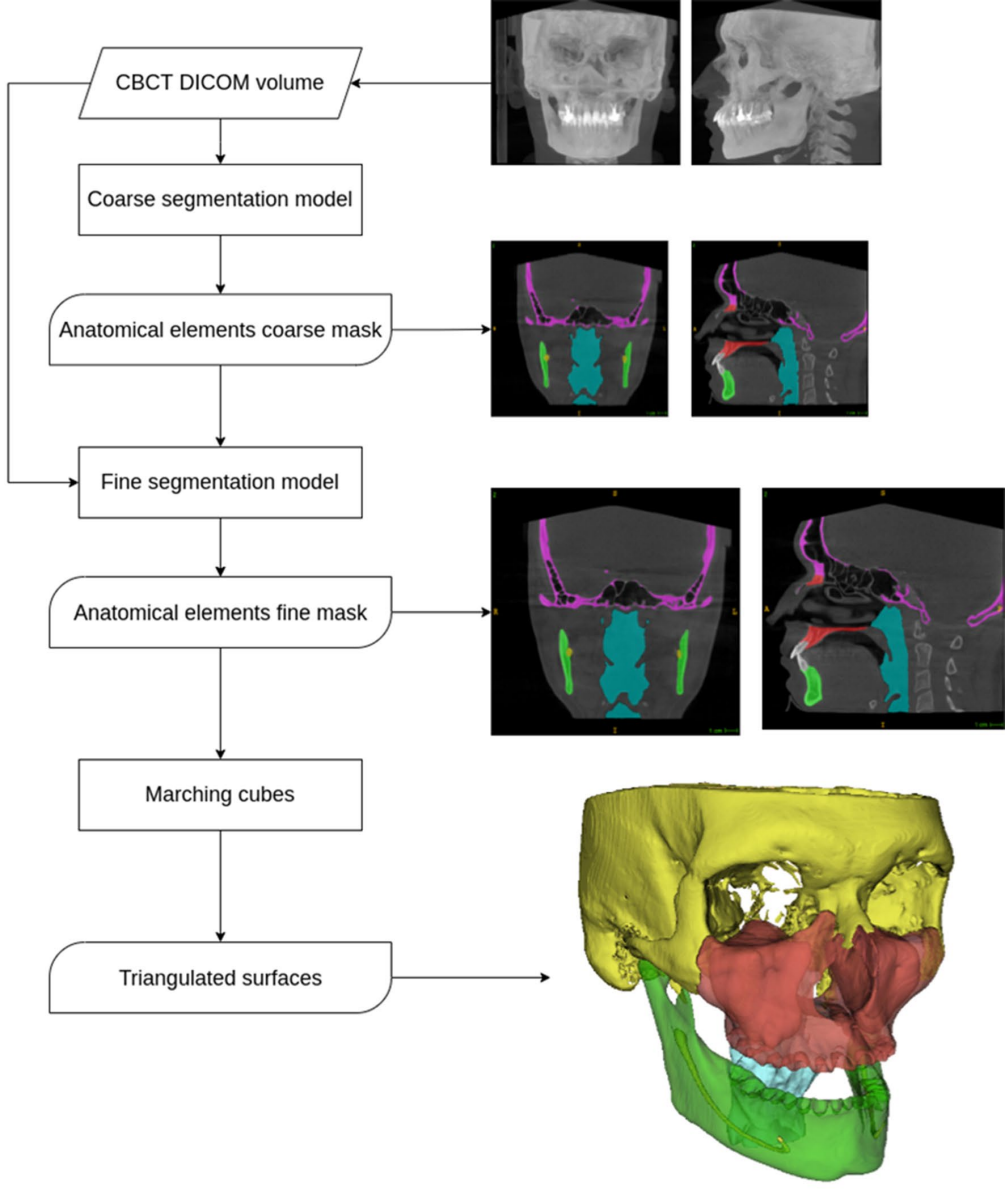
Inference algorithm of our study.

At test time the patch-based approach is known to produce the predictions of a worse quality near the borders of the output patch. To alleviate this issue, we perform inference in overlapping patches and aggregate the predictions with weights which make the center voxel of an output patch contribute more to the final result than its borders. We set the patches’ overlap to 16 (Fig. 1).

### Patient test dataset

To estimate the generalizability of our model, a retrospective patient CBCT dataset from Dentomaxillofacial Radiology Department at Near East University was used. A power analysis was conducted with a statistical power of 90%, a significance level of 0.05 α, and a probability of type II error of 0.2 β. A minimum number of 82 CBCT images for both control and OSA groups were required according to the power analysis.

Hence, our study was conducted with randomly selected artifact-free 100 OSA and 100 control CBCT images existing in our faculty's database. All patients provided their informed consent before irradiation, and the consent forms were reviewed and approved by the institutional review board of the faculty. Exclusion criteria were evident skeletal asymmetries, cleft palate, cleft lip, current ongoing orthodontic treatment, and any teeth that overlie the apical region of the incisors.

The dataset of a previous study^39^ of ours is used in this study "CBCT records of 200 patients (100 images of OSA patients and 100 images of the control group) were retrospectively collected and analyzed along with the polysomnography records and body mass index (BMI) of OSA patients at the Department of Allergy, Sleep and Respiratory Diseases. AHI is the number of apnea + hypopnea seen each hour during sleep. Sleep apnea severity was evaluated in 4 different subtypes minimal, mild, moderate, and severe. Patients with Apnea–Hypopnea Index (AHI) value lower than 5 were classified as a minimal group while patients with AHI values between 5–15, 15–30, and more than 30 were classified as mild, moderate, and severe, respectively. 100 OSA patients had symptoms of this disease and evaluation of these patients was accomplished by a standardized program at the Department of Allergy, Sleep and Respiratory Diseases, which also consists of anthropometric measurements, dental examination, CBCT, and polysomnography. Polysomnography uses various methods like electroencephalography, electromyogram, electro-oculography, respiratory effort measurement, airflow measurement, and snoring^29^. Control (non-OSA) patients had none of the clinical findings of the OSA patients such as snoring, dyspnea, witnessed apnea, coughing, or daytime sleepiness. So their images were used as a control group. The mean age for OSA patients was 53.2 years and for non-OSA patients was 46.4 years. Principles characterized in the Declaration of Helsinki were applied during the protocol of study along with modifications and revisions.

CBCT images of the test group were obtained by NewTom 3 G Quantitive Radiology s.r.l., (NewTom, Verona, Italy). CBCT records for non-OSA patients had been taken for implant planning, evaluation of impacted teeth, and prosthodontic and orthodontic purposes. Patients with osteoporosis, skeletal asymmetries, and medication-related bony alterations were excluded from the study.

### Ground truth segmentation process

All CBCT data were exported as DICOM files and then anonymized. The axial, coronal, and sagittal slices were oriented to ensure a proper evaluation. The axial slices were aligned with maintaining the palate line and the ground perpendicular to each other. Coronal slices were oriented by aligning the both orbits and midline of the head parallel to the ground and the sagittal slices were aligned with the linear orientation of the ANS and PNS.

All CBCT images had been segmented before our study to be used for diagnosis, pharyngeal airway evaluations, and surgical planning using InVivo 5.1.2 (*Anatomage Inc., San Jose, CA, USA*). DICOM files of the axial CBCT images were exported with a 512 × 512 matrix and were imported to InVivo 5.1.2. In this software, the evaluation of the pharyngeal airway can be measured by both automatic thresholding and manual tracing with semiautomatic thresholding.

The pharyngeal airway is originated from the nasopharynx and the oropharynx. To assess the borders of the oropharyngeal airway volume, the ANS-PNS plane which extends to the wall of the pharynx was determined as the superior border and the most inferior-anterior point of the 2nd cervical vertebrae which is parallel to the superior border was determined as the lower border of the oropharyngeal airway. Since the superior border of the oropharyngeal airway is also the lower border of the nasopharyngeal airway, a line perpendicular from the PNS to the palatal plane is drawn to form the anterior border of the nasopharyngeal airway. The Sum of the nasopharyngeal airway and oropharyngeal airway is calculated with both manual tracing with semi-automatic thresholding and automatic thresholding in InVivo 5.1.2. viewer. S.A. and A.K. observed the CBCT images twice with a week interval to avoid any intra-observer disagreement for ground truth measurement.

For automatic thresholding, the software itself detects the pharyngeal airway volume, area narrow point area, and measures the narrow point automatically.

The manual tracing with semiautomatic thresholding was done by cropping the airway using the "edit masks" feature and the connection with the outer air was cropped in each slice with the segmentation tools. The "region growing" tool was used to split the segmentation produced by thresholding into several objects and to remove floating pixels and the pharyngeal airway volume and area were calculated using the “calculate 3D” tool feature of the software.

### 3D U-net architecture framework (AI model)

Our approach is automatic segmentation focusing on the regions of interest: the external surface of the bones, teeth, and airways. This process results in 5 segmentation masks the upper skull, the mandible, maxillary teeth, mandibular teeth, and the airways. We performed a series of trials to choose the best training configuration. Following, the generated STL files were downloaded and imported to 3rd party software for volumetric pharyngeal airway measurements (*3-Matic Version 15, Materialise*).

### Statistical analysis

Statistical analysis was performed using SPSS 22.0 software (*SPSS Inc., Chicago, IL, USA*). Due to the non-normal distribution of the data, the Mann–Whitney U test was used for comparisons between paired groups, and the Kruskall Wallis H test was used for comparisons between three or more groups. The significance level was set as 0.05 and it was stated that there was a significant difference in the case of *p* < 0.05, and no significant difference in the case of *p* > 0.05. Interclass correlation coefficient (ICC) analysis with a two-way mixed model was performed. It was assumed that ICC values greater than 0.75 would guarantee good reliability and ICC values greater than 0.90 would guarantee excellent reliability between observers.

## Results

There was no statistically significant difference in airway volume (cc) measurement difference between the manual measurement and DC in any of the OSA severity subtypes (*p* > 0.05). *p* values were 0.052, 0.942, 0.642, and 0.207 for the minimal, mild, moderate, and severe OSA groups, respectively (Table 1). Statistical analysis showed excellent ICC (ICC > 0.90) for all inter-evaluator assessments. ICC values were 0.954 for manual and automatic segmentation, 0.956 for DC and automatic segmentation, 0.972 for DC and manual segmentation.

**Table 1:** Comparison of the airway volume measurements of Diagnocat and manual technique in patients with different OSA severities.

OSA severity	The technique (subgroup)	Mean	Median	Min	Max	SD	Mann Whitney U		
							Mean rank	U	*p*
**Airway volume (cc)**									
Minimal OSA	Manual	21.18	21.03	11.81	34.87	6.49	31.67	252	0.052
	Diagnocat	17.73	16.20	7.60	29.60	6.16	23.33		
	Total	19.45	19.66	7.60	34.87	6.51			
Mild OSA	Manual	18.32	17.72	7.64	28.46	6.23	19.63	178	0.942
	Diagnocat	18.11	17.60	7.70	29.50	6.18	19.37		
	Total	18.22	17.66	7.64	29.50	6.12			
Moderate OSA	Manual	22.42	22.55	9.69	35.53	7.45	22.38	202	0.642
	Diagnocat	21.42	21.20	6.60	35.30	6.96	20.62		
	Total	21.92	21.92	6.60	35.53	7.14			
Severe OSA	Manual	19.21	17.85	7.34	34.97	7.54	36.48	446	0.207
	Diagnocat	16.79	15.20	5.60	29.60	6.73	30.52		
	Total	18.00	15.72	5.60	34.97	7.20			

### Measurements for non-OSA patients

There was no statistically significant difference in narrowest points (mm), airway area (mm^2^), and total airway volume (cc) measurements between the manual measurements and DC measurements in non-OSA patients. *p* values were 0.346, 0.111 and 0.667, respectively. The mean value for the narrowest distance was found 5.96 mm with the manual measurement and 5.70 mm with DC. The mean value for the airway area was found 883.41 mm^2^ with the manual measurement and 930.02 mm^2^ with DC. The mean value for the total airway volume was found 17.95 cc with the manual measurement, 17.96 cc with the automatic technique, and 18.50 cc with DC (Table 2).

**Table 2:** Comparison of airway volume, airway area, and narrowest line of the airway measurements of Diagnocat, manual technique, and automatic technique in patients without OSA.

	Non-OSA patients									
	n	Mean	Median	Minimum	Maximum	SD	Mean Rank	U	*p*	Test
**Narrowest points (mm)**										
Manual	100	5.96	5.58	1.89	13.30	2.07	104.36	4614.5	0.346	Mann–Whitney U
Diagnocat	100	5.70	5.41	1.69	14.56	2.10	96.65			
Total	200	5.83	5.46	1.69	14.56	2.08				
**Airway area (mm**^**2**^**)**										
Manual	100	883.41	856.73	437.65	1576.88	212.92	93.97	4347	0.111	Mann–Whitney U
Diagnocat	100	930.02	909.00	597.33	1694.00	201.18	107.03			
Total	200	906.71	895.67	437.65	1694.00	207.93				
**Volume (cc)**										
Manual	100	17.95	17.70	4.90	34.10	5.45	146.12	0.811	0.667	Kruskall-Wallis H
Diagnocat	100	18.50	18.40	5.50	35.20	5.63	156.71			
Automatic	100	17.96	18.20	4.80	32.80	5.41	148.68			
Total	300	18.14	18.00	4.80	35.20	5.48				

### Measurements for OSA patients

There was, also, no statistically significant difference in narrowest points (mm), airway area (mm^2^), and total airway volume (cc) measurements between the manual measurements and DC measurements in OSA patients. *p* values were 0.931, 0.305 and 0.139, respectively. The mean value for the narrowest distance was found 6.31 mm with the manual measurement and 6.10 mm with DC. The mean value for the airway area was found 1057.59 mm^2^ with the manual measurement and 1013.90 mm^2^ with DC. The mean value for the total airway volume was found 19.63 cc with the manual measurement, 18.27 cc with the automatic measurement, and 20.25 cc with DC (Table 3).

**Table 3:** Comparison of airway volume, airway area, and narrowest line of the airway measurements of Diagnocat, manual technique, and automatic technique in patients with OSA.

	OSA patients									
	n	Mean	Median	Minimum	Maximum	SD	Mean Rank	U	*p*	Test
**Narrowest points (mm)**										
Manual	100	6.31	5.86	1.48	23.08	3.42	100.14	4964	0.931	Mann–Whitney U
Diagnocat	100	6.10	5.76	1.01	19.90	2.50	100.86			
Total	200	6.20	5.78	1.01	23.08	2.99				
**Airway area (mm**^**2**^**)**										
Manual	100	1057.59	1033.07	598.85	1731.64	244.38	104.70	4580	0.305	Mann–Whitney U
Diagnocat	100	1013.90	989.81	466.52	1670.22	256.57	96.30			
Total	200	1035.74	1001.13	466.52	1731.64	250.88				
**Volume (cc)**										
Manual	100	19.63	19.05	7.40	35.30	6.90	153.05	3.9	0.139	Kruskall-Wallis H
Diagnocat	100	20.25	19.83	7.34	35.53	7.08	161.21			
Automatic	100	18.27	17.50	5.60	35.30	6.65	137.24			
Total	300	19.38	18.55	5.60	35.53	6.91				

## Discussion

According to our review of the literature, this study is the first study that automatically measured the pharyngeal airway in OSA patients. However, manual measurements of the pharyngeal airway in OSA patients and automatic measurements of the pharyngeal airway in non-OSA patients are present in the literature. Since the studies demonstrated that the pharyngeal airway volume is significantly lower and the morphology is dissimilar in oral breathers than in nasal breathers, deep learning algorithms which concentrate on airway volume measurements should be trained and tested with various data. In orthodontics, airway volume and the underlying factors play a crucial role before orthognathic surgery planning, analyzing the airway volume is indispensable to understanding the oral and pharyngeal adjustments to respiratory conditions^12,40–42^.

Although there was no statistically significant difference in total airway volume measurements between the manual measurements, automatic measurements, and DC measurements in non-OSA and OSA patients, we evaluated the output images to understand why the mean value for the total airway was higher in DC measurement. It was seen that the DC algorithm also measures the epiglottis volume and the posterior nasal aperture volume due to the low soft-tissue contrast in CBCT images and that leads to higher values in airway volume measurement.

The mean total airway volume difference between automatic measurement and manual measurement in non-OSA patients was just 0.01 cc, however, it was 1.36 cc in OSA patients. Output images were again compared and it was seen that there were voxel loss sites at the posterior nasal aperture border in the automatic measurement group.

Various authors measured the airway in adults and according to their findings, the difference between their manual measurements has occurred due to the “human factor” and different software that is used for the measurements^12,43,44^. Following an extensive literature research, we have given the ICC values of 5 studies that compared the segmentation of AI and ground truth in Table 4. ICC values were reported as 0.899 by Zhang et al.^45^, 0.977 by Leonardi et al.^46^, 0.985 by Sin et al.^12^, 0.986 by Park et al.^47^. Shujaat et al.^48^ provided precision, recall, accuracy, dice, intersection over union values in their study as 0.97 ± 0.01, 0.96 ± 0.03 1.00 ± 0.00 0.97 ± 0.02 and 0.93 ± 0.03, respectively. In our study, the ICC value between the ground truth and DC was 0.972 which indicates that an excellent reliability was present. As the previous studies which aimed to segment and measure the pharyngeal airway volume, we also achieved a high ICC value which evidently shows that well-trained deep learning algorithms can successfully segment the pharyngeal airway.

**Table 4:** Comparative table of the studies using deep learning algorithms for pharyngeal airway volume segmentation and measurement.

Authors	Year	Title	Modality	Softwares	Interclass correlation coefficient	Intersection over union (IoU)
Zhang et al.	2019	A new segmentation algorithm for measuring CBCT images of nasal airway: a pilot study	CBCT	Airway Segmentor, MIMICS 19.0, InVivo 5	0.899	
Leonardi et al.	2020	Fully automatic segmentation of sinonasal cavity and pharyngeal airway based on convolutional neural networks	CBCT	Own model	0.977	
Sin et al.	2021	A deep learning algorithm proposal to automatic pharyngeal airway detection and segmentation on CBCT images	CBCT	Own model	0.985	
Park et al.	2021	Deep learning based airway segmentation using key point prediction	CBCT	Own model	0.986	
Shujaat et al.	2021	Automatic segmentation of the pharyngeal airway space with convolutional neural network	CBCT, MSCT	Own model	–	0.93
This study	2022	AI-based automatic segmentation of craniomaxillofacial anatomy from CBCT scans for automatic detection of pharyngeal airway evaluations in OSA patients	CBCT	Diagnocat, InVivo 5	Manual-Automatic 0.954	
					DC-Automatic 0.956	
					DC-Manual 0.972	

In our study, we had an imaging modality-related limitation, which was caused by the limited soft-tissue contrast of the CBCT units. Arranging a precise Hounsfield Unit (HU) threshold in the segmentation process is not possible with CBCT units since HUs are not applicable for them^49^. This limitation might affect the airway volume measurements as they have affected our segmentation process. The inconsistent head position of the patients, tongue, and breathing positions also cause errors in volumetric measurement, thus, scannings with controlling these possible limitations are required^47^.

AI is widely known for its functions in image recognition, computer-aided diagnosis, and decision-making algorithms. Given that 90% of the clinical data is medical images, AI can collaborate with the Internet of Things (IoT) to make health care more advanced with the remote diagnosis that can accelerate the diagnosis and treatment phases^50–52^. Activating this potential collaboration for OSA patients would significantly reduce the effort and time required for the initial diagnosis and follow-up of these patients.
